# (*E*)-2-(2-Nitro­prop-1-en­yl)thio­phene

**DOI:** 10.1107/S1600536811010622

**Published:** 2011-03-26

**Authors:** Zhao-Bo Li, Li-Li Shen, Jia-Jia Li

**Affiliations:** a Hangzhou Minsheng Pharmaceutical Group Co. Ltd, Hangzhou 310000, People’s Republic of China; b Zhejiang University of Technology, Hangzhou 310000, People’s Republic of China; c Hangzhou Radio & TV University, Hangzhou 310000, People’s Republic of China

## Abstract

The title compound, C_7_H_7_NO_2_S, adopts an *E* conformation about the C=C bond. The torsion angle C=C—C—C is −177.7 (3)°. The crystal structure features weak inter­molecular by C—H⋯O inter­actions.

## Related literature

For the use of nitro­alkenes as organic inter­mediates, see: Ballini & Petrini (2004 ▶); Berner *et al.* (2002 ▶); Ono (2001 ▶). 
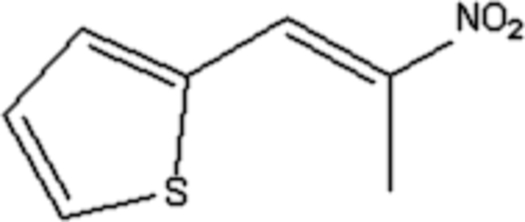


## Experimental

### 

#### Crystal data

C_7_H_7_NO_2_S
*M* *_r_* = 169.20
Monoclinic, 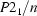

*a* = 6.7545 (6) Å
*b* = 16.6940 (13) Å
*c* = 7.4527 (4) Å
β = 110.640 (7)°
*V* = 786.42 (10) Å^3^
*Z* = 4
Mo *K*α radiation
μ = 0.36 mm^−1^
*T* = 296 K
0.31 × 0.18 × 0.17 mm

#### Data collection

Rigaku R-AXIS RAPID diffractometer
Absorption correction: multi-scan (*ABSCOR*; Higashi, 1995 ▶) *T*_min_ = 0.879, *T*_max_ = 0.942
5936 measured reflections
1362 independent reflections
971 reflections with *I* > 2σ(*I*)
*R*_int_ = 0.035

#### Refinement

*R*[*F* ^2^ > 2σ(*F* ^2^)] = 0.049
*wR*(*F* ^2^) = 0.170
*S* = 1.00
1362 reflections
102 parameters
H-atom parameters constrained
Δρ_max_ = 0.39 e Å^−3^
Δρ_min_ = −0.33 e Å^−3^

### 

Data collection: *PROCESS-AUTO* (Rigaku, 2006 ▶); cell refinement: *PROCESS-AUTO*; data reduction: *CrystalStructure* (Rigaku Americas and Rigaku, 2007 ▶); program(s) used to solve structure: *SHELXS97* (Sheldrick, 2008 ▶); program(s) used to refine structure: *SHELXL97* (Sheldrick, 2008 ▶); molecular graphics: *ORTEP-3 for Windows* (Farrugia, 1997 ▶); software used to prepare material for publication: *WinGX* (Farrugia, 1999 ▶).

## Supplementary Material

Crystal structure: contains datablocks I, global. DOI: 10.1107/S1600536811010622/jh2274sup1.cif

Structure factors: contains datablocks I. DOI: 10.1107/S1600536811010622/jh2274Isup2.hkl

Additional supplementary materials:  crystallographic information; 3D view; checkCIF report

## Figures and Tables

**Table 1:** Hydrogen-bond geometry (Å, °)

*D*—H⋯*A*	*D*—H	H⋯*A*	*D*⋯*A*	*D*—H⋯*A*
C6—H6⋯O2^i^	0.93	2.60	3.511 (5)	168

Symmetry code: (i) 
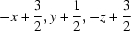
.

**Fig. 1.**
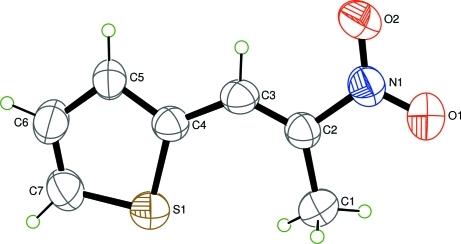
The asymmetric unit of the title compound with the atomic labeling scheme; displacement ellipsoids are drawn at the 50% probability level.

**Fig. 2.**
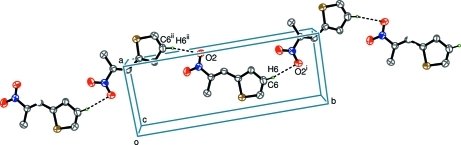
The view of intermolecular interaction illustrated as dash lines.

## supplementary crystallographic information

### Comment

Nitroalkenes are important organic intermediates, since they can be converted to synthetically useful N– and O-containing organic molecules, such as amines, aldehydes, carboxylic acids, or denitrated compounds (Ono, 2001; Berner *et al.*, 2002; Ballini & Petrini, 2004). As a contribution in this field, we have synthesized a series of nitroalkenes by employing benzaldehydes and nitroethane. We report here one of this nitroalkenes, *i.e.* the crystal structure of the title compound. The C2═C3 bond involves the *E* configuration with the C2—C3—C4—C5 torsion angle of 177.71 (3)° (Fig. 1). The atoms of the thiophene ring are coplanar. The conformation of (I) is stabilized by weak intermolecular by C6—H6···O2' interaction (Fig. 2 and Table 1).

### Experimental

To a solution of thiophene-2-carbaldehyde (50 mmol) in AcOH (25 mL), nitroethane (75 mmol) was added, followed by butylamine (100 mmol, 7.4 mL). The mixture was sonicated at 60 °C, until GC showed full conversion of the aldehyde. The mixture was poured into ice water, the precipitate was filtered off, washed with water and recrystallized from EtOH/EtOAc to give the product. Single crystals were obtained by slow evaporation of an cyclohexane-EtOAc solution (10:1, *v*/*v*).

### Refinement

All H atoms were placed in calculated positions and refined using a riding model, with C—H = 0.93–0.96 Å, and with *U*_iso_(H) = 1.2 *U*_eq_(C) or 1.5 *U*_eq_(C) for methyl H atoms.

### Crystal data

**Table:**

C_7_H_7_NO_2_S	*F*(000) = 352
*M**_r_* = 169.20	*D*_x_ = 1.429 Mg m^−^^3^
Monoclinic, *P*2_1_/*n*	Mo *K*α radiation, λ = 0.71073 Å
Hall symbol: -P 2yn	Cell parameters from 3534 reflections
*a* = 6.7545 (6) Å	θ = 3.2–27.4°
*b* = 16.6940 (13) Å	µ = 0.36 mm^−^^1^
*c* = 7.4527 (4) Å	*T* = 296 K
β = 110.640 (7)°	Prism, yellow
*V* = 786.42 (10) Å^3^	0.31 × 0.18 × 0.17 mm
*Z* = 4	

### Data collection

**Table:**

Rigaku R-AXIS RAPID diffractometer	1362 independent reflections
Radiation source: rolling anode	971 reflections with *I* > 2σ(*I*)
graphite	*R*_int_ = 0.035
Detector resolution: 10.00 pixels mm^-1^	θ_max_ = 25.0°, θ_min_ = 3.2°
ω scans	*h* = −7→8
Absorption correction: multi-scan (*ABSCOR*; Higashi, 1995)	*k* = −19→19
*T*_min_ = 0.879, *T*_max_ = 0.942	*l* = −8→8
5936 measured reflections	

### Refinement

**Table:**

Refinement on *F*^2^	Secondary atom site location: difference Fourier map
Least-squares matrix: full	Hydrogen site location: inferred from neighbouring sites
*R*[*F*^2^ > 2σ(*F*^2^)] = 0.049	H-atom parameters constrained
*wR*(*F*^2^) = 0.170	*w* = 1/[σ^2^(*F*_o_^2^) + (0.0837*P*)^2^ + 0.8184*P*] where *P* = (*F*_o_^2^ + 2*F*_c_^2^)/3
*S* = 1.00	(Δ/σ)_max_ = 0.001
1362 reflections	Δρ_max_ = 0.39 e Å^−^^3^
102 parameters	Δρ_min_ = −0.33 e Å^−^^3^
0 restraints	Extinction correction: *SHELXL97* (Sheldrick, 2008), Fc^*^=kFc[1+0.001xFc^2^λ^3^/sin(2θ)]^-1/4^
Primary atom site location: structure-invariant direct methods	Extinction coefficient: 0.0067 (6)

### Special details

**Table:**

Geometry. All e.s.d.'s (except the e.s.d. in the dihedral angle between two l.s. planes) are estimated using the full covariance matrix. The cell e.s.d.'s are taken into account individually in the estimation of e.s.d.'s in distances, angles and torsion angles; correlations between e.s.d.'s in cell parameters are only used when they are defined by crystal symmetry. An approximate (isotropic) treatment of cell e.s.d.'s is used for estimating e.s.d.'s involving l.s. planes.
Refinement. Refinement of *F*^2^ against ALL reflections. The weighted *R*-factor *wR* and goodness of fit *S* are based on *F*^2^, conventional *R*-factors *R* are based on *F*, with *F* set to zero for negative *F*^2^. The threshold expression of *F*^2^ > σ(*F*^2^) is used only for calculating *R*-factors(gt) *etc*. and is not relevant to the choice of reflections for refinement. *R*-factors based on *F*^2^ are statistically about twice as large as those based on *F*, and *R*- factors based on ALL data will be even larger.

### Fractional atomic coordinates and isotropic or equivalent isotropic displacement parameters (Å^2^)

**Table:**

	*x*	*y*	*z*	*U*_iso_*/*U*_eq_	
S1	0.21787 (15)	0.56891 (6)	0.61453 (16)	0.0684 (5)	
C4	0.4914 (5)	0.5654 (2)	0.7041 (5)	0.0496 (8)	
C3	0.6192 (5)	0.4976 (2)	0.7933 (4)	0.0500 (8)	
H3	0.7644	0.5067	0.8373	0.060*	
N1	0.7349 (5)	0.36806 (18)	0.9203 (4)	0.0584 (8)	
O2	0.9190 (4)	0.39008 (17)	0.9632 (5)	0.0775 (9)	
C2	0.5624 (5)	0.4238 (2)	0.8227 (5)	0.0493 (8)	
O1	0.6885 (5)	0.30107 (17)	0.9578 (5)	0.0832 (9)	
C5	0.5793 (6)	0.64019 (19)	0.6781 (5)	0.0512 (8)	
H5	0.7230	0.6515	0.7150	0.061*	
C1	0.3450 (6)	0.3888 (2)	0.7708 (6)	0.0652 (10)	
H1A	0.2975	0.3941	0.8775	0.098*	
H1B	0.3487	0.3332	0.7398	0.098*	
H1C	0.2496	0.4168	0.6621	0.098*	
C7	0.2176 (6)	0.6653 (2)	0.5463 (6)	0.0698 (11)	
H7	0.0948	0.6947	0.4869	0.084*	
C6	0.4120 (6)	0.6951 (2)	0.5863 (6)	0.0675 (11)	
H6	0.4365	0.7475	0.5568	0.081*	

### Atomic displacement parameters (Å^2^)

**Table:**

	*U*^11^	*U*^22^	*U*^33^	*U*^12^	*U*^13^	*U*^23^
S1	0.0499 (6)	0.0587 (7)	0.0895 (8)	−0.0017 (4)	0.0158 (5)	0.0006 (5)
C4	0.0447 (19)	0.051 (2)	0.0507 (19)	−0.0015 (14)	0.0131 (15)	−0.0040 (14)
C3	0.0419 (17)	0.052 (2)	0.052 (2)	−0.0039 (14)	0.0124 (15)	−0.0078 (15)
N1	0.0557 (19)	0.0507 (19)	0.0660 (19)	0.0060 (14)	0.0180 (15)	0.0000 (14)
O2	0.0474 (15)	0.0661 (18)	0.107 (2)	0.0055 (12)	0.0125 (15)	0.0016 (16)
C2	0.0468 (18)	0.0464 (19)	0.0532 (19)	0.0018 (14)	0.0157 (15)	−0.0040 (14)
O1	0.079 (2)	0.0533 (17)	0.118 (3)	0.0078 (14)	0.0349 (18)	0.0175 (16)
C5	0.056 (2)	0.0418 (18)	0.0499 (19)	0.0022 (14)	0.0116 (15)	0.0006 (14)
C1	0.054 (2)	0.056 (2)	0.082 (3)	−0.0085 (17)	0.0198 (19)	0.0007 (19)
C7	0.058 (2)	0.058 (2)	0.083 (3)	0.0100 (18)	0.012 (2)	0.002 (2)
C6	0.074 (3)	0.047 (2)	0.076 (3)	−0.0032 (18)	0.019 (2)	0.0038 (18)

### Geometric parameters (Å, °)

**Table:**

S1—C7	1.688 (4)	C2—C1	1.499 (5)
S1—C4	1.730 (3)	C5—C6	1.429 (5)
C4—C5	1.425 (5)	C5—H5	0.9300
C4—C3	1.436 (5)	C1—H1A	0.9600
C3—C2	1.331 (5)	C1—H1B	0.9600
C3—H3	0.9300	C1—H1C	0.9600
N1—O1	1.220 (4)	C7—C6	1.336 (5)
N1—O2	1.226 (4)	C7—H7	0.9300
N1—C2	1.469 (4)	C6—H6	0.9300
			
C7—S1—C4	92.09 (18)	C4—C5—H5	125.4
C5—C4—C3	122.8 (3)	C6—C5—H5	125.4
C5—C4—S1	110.9 (2)	C2—C1—H1A	109.5
C3—C4—S1	126.3 (3)	C2—C1—H1B	109.5
C2—C3—C4	130.0 (3)	H1A—C1—H1B	109.5
C2—C3—H3	115.0	C2—C1—H1C	109.5
C4—C3—H3	115.0	H1A—C1—H1C	109.5
O1—N1—O2	122.3 (3)	H1B—C1—H1C	109.5
O1—N1—C2	118.1 (3)	C6—C7—S1	113.0 (3)
O2—N1—C2	119.6 (3)	C6—C7—H7	123.5
C3—C2—N1	116.3 (3)	S1—C7—H7	123.5
C3—C2—C1	129.2 (3)	C7—C6—C5	114.7 (4)
N1—C2—C1	114.5 (3)	C7—C6—H6	122.7
C4—C5—C6	109.3 (3)	C5—C6—H6	122.7
			
C7—S1—C4—C5	−0.3 (3)	O1—N1—C2—C1	−3.2 (5)
C7—S1—C4—C3	179.8 (3)	O2—N1—C2—C1	177.6 (3)
C5—C4—C3—C2	−177.7 (3)	C3—C4—C5—C6	−179.8 (3)
S1—C4—C3—C2	2.1 (6)	S1—C4—C5—C6	0.3 (4)
C4—C3—C2—N1	−179.7 (3)	C4—S1—C7—C6	0.2 (4)
C4—C3—C2—C1	0.0 (6)	S1—C7—C6—C5	−0.1 (5)
O1—N1—C2—C3	176.6 (3)	C4—C5—C6—C7	−0.2 (5)
O2—N1—C2—C3	−2.6 (5)		

### Hydrogen-bond geometry (Å, °)

**Table:**

*D*—H···*A*	*D*—H	H···*A*	*D*···*A*	*D*—H···*A*
C6—H6···O2^i^	0.93	2.60	3.511 (5)	168

Symmetry codes: (i) −*x*+3/2, *y*+1/2, −*z*+3/2.
